# Mapping neuro-disabilities and their dimensions among under 5 years of age children in the southern agricultural corridor of Tanzania: a preliminary baseline survey

**DOI:** 10.3389/fpsyg.2024.1426870

**Published:** 2025-01-17

**Authors:** Peter M. Chilipweli, Namanya Basinda, Paul Alikado Sabuni, Fredy Hyera, Upendojackline Liana, Titus Robert Leeyio, Elias C. Nyanza, Awerasia Vera Ngowi

**Affiliations:** ^1^Department of Community Medicine, School of Public Health, Catholic University of Health and Allied Sciences (CUHAS), Mwanza, Tanzania; ^2^Department of Epidemiology, Biostatistics, and Behavior Sciences, School of Public Health, Catholic University of Health and Allied Sciences (CUHAS), Mwanza, Tanzania; ^3^Research and Consultancy Unit, Bugando Medical Center (BMC), Mwanza, Tanzania; ^4^School of Medicine, Catholic University of Health and Allied Sciences (CUHAS), Mwanza, Tanzania; ^5^Department of Environmental Occupational Health and GIS, School of Public Health, Catholic University of Health and Allied Sciences (CUHAS), Mwanza, Tanzania; ^6^Department of Occupational and Environmental Health, School of Public Health, Muhimbili University of Health and Allied Sciences (MUHAS), Dar es Salaam, Tanzania

**Keywords:** neuro-disability, autism spectrum disorder, attention-deficit/hyperactivity disorder, prevalence, targeted interventions

## Abstract

**Background:**

Neuro-disabilities involve impairments of the nervous system, affecting brain development and functioning. Due to limited scientific data on neuro-disabilities in Tanzania, this study examines maternal characteristics in high-risk areas, such as the Southern Agricultural Growth Corridor (SAGCOT).

**Methods:**

This cross-sectional study sampled 286 children aged 0–5 years and their mothers in the SAGCOT region. Each mother selected the youngest child within the specified age range. Multi-stage sampling was used to choose clusters and areas for the study. The Malawi Developmental Assessment Tool (M-DAT) was used to assess the children’s developmental levels. Descriptive analysis determined distribution patterns, while multivariate analyses were performed to identify significant factors. Modified Poisson regression with robust standard errors estimated prevalence ratios (PRs) and their 95% confidence intervals (CIs).

**Results:**

The study included 286 mother–child pairs from four clusters: Ihemi, Kilombero, Ludewa, and Mbarali. The children’s median age was 24 months, with the majority aged between 13 and 48 months. Boys constituted a slight majority (59.4%). The majority of mothers were married (72.4%), had primary education (56.6%), and were engaged in non-farming occupations (56.3%). Neurodevelopmental assessments revealed that 11.2% of children were fully developed, while 88.8% exhibited development delays. Adjusted prevalence ratios (aPR) with 95% confidence intervals identified significant associations between developmental domains and demographic variables such as age, gender, marital status, and maternal occupation.

**Conclusion:**

The study highlights a high prevalence of neuro-disability among children in Tanzania’s southern corridor, revealing disparities across regions and the impact of factors such as gender and marital status. Targeted interventions are essential to address these developmental challenges effectively and promote optimal child development and wellbeing.

## 1 Background

### 1.1 Introduction

Neuro-disabilities refer to conditions that impair the nervous system, affecting neurological pathways and overall functioning. These conditions include cerebral palsy, autism, attention deficit hyperactivity disorder (ADHD), and epilepsy (Autism and Developmental Disabilities Monitoring Network Surveillance Year 2008 Principal Investigators and Centers for Disease Control and Prevention, 2012). Neuro-disabilities are lifelong disorders characterized by difficulties in cognitive functioning, intellectual disability, social-communication domains, and the presence of restricted and/or repetitive interests and behaviors.

ADHD is a neurodevelopmental disorder of childhood onset, marked by persistent inattention, hyperactivity, and impulsivity, which impair a child or adolescent’s functioning. ADHD is the most prevalent psychiatric disorder among youth. In the United States, the diagnosis rates of ADHD among children and adolescents have increased significantly over two decades, rising from 6.1 to 10.2% between 1997 and 2016 (Awadu et al., 2022).

Autism Spectrum Disorder (ASD) is another serious developmental disorder, typically diagnosed before the age of three, imposing a high economic burden (Bakare et al., 2012). Autism manifests before 3 years of age in the majority of children and is characterized by a high degree of heterogeneity in phenotypic manifestations. This heterogeneity is associated with wide variability in levels of intellectual and language development and intra-individual discrepancies in cognitive profiles (Baron-Cohen et al., 2009).

According to the World Health Organization (WHO), currently, 1 in 160 children is born with neuro-disabilities (Chaste and Leboyer, 2012). Evidence suggests a rapid increase in the prevalence of ASD in recent years, with boys being four to five times more affected than girls (Boyle et al., 2011). This gender disparity is also evident in studies like the one conducted in Alabama, which found significantly higher ASD prevalence estimates among boys than girls (Chaste and Leboyer, 2012).

Neuro-disabilities prevalence estimates have risen from 6.7 per 1,000 children aged 8 years at Autism and Developmental Disabilities Monitoring (ADDM) Network sites in surveillance years 2000 and 2002 to 18.5 in surveillance year 2016 (Bakare et al., 2012; Chaste and Leboyer, 2012; Chilipweli et al., 2021; Fombonne, 2010; Gunier et al., 2017; Honda et al., 2005; Honda et al., 1996). Over time, the proportion of children with ASD who also have intellectual disability has decreased from approximately half in 2000 and 2002 to one-third in 2016 (Honda et al., 1996).

In the North Africa and Middle East region, a study by Meimand et al. (Hoshino et al., 1982) found that the number of new neuro-disability cases increased by approximately 1.8% from 1990 to 2019, reaching 45,002 cases in 2019, which accounted for 7.5% of the global total of new neuro-disability cases that year. The study also revealed that boys’ age-standardized incidence rate (ASIR) was 2.9 times higher than that for girls in 2019.

In sub-Saharan Africa, the prevalence of neuro-disabilities among children with intellectual disabilities has increased by about 0.7% over the past three decades. A study conducted in Nigeria observed that 11.4% of children with intellectual disabilities had neuro-disabilities, with a male-to-female ratio of 4:1. These findings collectively suggest a gradual increase in the prevalence of neuro-disabilities in Africa.

Various risk factors for neuro-disabilities have been identified, including genetics, advanced parental age, prenatal factors such as environmental exposures and gestational diabetes, birth complications, parental education level, socioeconomic status, maternal stress, immune system dysfunction, and neurological factors such as differences in brain development and neurotransmitter abnormalities (Awadu et al., 2022; Chaste and Leboyer, 2012; Huerta et al., 2012).

There is limited information available on the prevalence and characteristics of neuro-disabilities among children in Tanzania, as most studies have focused on the knowledge, prevalence, and care of children with ASD (Chilipweli et al., 2021; Isaksen et al., 2012). These studies often overlook the broader neuro-disabilities and their association with maternal characteristics, particularly in high-risk areas such as the Southern Agricultural Growth Corridor of Tanzania (SAGCOT). Therefore, this study aims to address this gap by mapping neuro-disabilities alongside maternal characteristics and determining the prevalence of autism in the SAGCOT.

## 2 Materials and methods

### 2.1 Study settings, study population, and study design

The study was conducted in the SAGCOT of Tanzania, located in the country’s Southern Zone. This region covers the Ruvuma, Iringa, Mbeya, Rukwa, Morogoro, Pwani, and Njombe regions. SAGCOT is characterized by extensive food production, reliance on irrigation schemes, and significant pesticide use. Despite its relative wealth and diverse occupational opportunities, the region has a high prevalence of malnutrition. This study focused on selected areas within the SAGCOT region based on the population density within agricultural clusters. Areas with higher populations were prioritized for inclusion. Consequently, the study was conducted in the Iringa-Ihemi, Morogoro-Kilombero, Mbeya-Mbarali, and Njombe-Ludewa clusters.

The study enrolled 286 mother-to-child pair participants who were estimated using the Kish Leslie formula (Jick and Kaye, 2003; Kish, 1965), which is for cluster sampling whereby the proportion of children with development effect used was 40.7%, referred from a study in Java in Indonesia (Gunier et al., 2017). The inclusion criteria were children 0–5 years old and their mothers, children with their mothers who were mentally fit, defined by their ability to comprehend questions asked and answer them correctly, simply an absence of disorder of the mind, and children who resided in a particular study area of the study. In contrast, the exclusion criteria were children with head trauma, children with chronic illness, and genetic and nutrition disorders.

### 2.2 Sampling and data collection

Sampling involved selecting the youngest child within the target age range from each mother in the SAGCOT region. The recruitment procedure was carefully designed to ensure a representative sample of the population within the SAGCOT. Multi-stage sampling was employed to select clusters and areas where the study was conducted. Purposive sampling was employed to select clusters. Thus, Ihemi, Kilombero, Ludewa, and Mbarali were selected based on the high number of participants in the agricultural cluster selected. One ward from each cluster was selected by a simple random sampling method using the lottery method, where the names of wards were coded on a piece of paper and randomly picked.

Probability proportional to size was conducted to select three villages of interest from each selected ward. Thus, a list of all landing sites in the village was obtained, with their respective population of members arranged in descending order, and the cumulative population was determined. The total population and the number of clusters or villages were used to calculate the sampling interval. Then, the first cluster was determined by getting a random number, and the cluster that had this selected random number was the first cluster selected. The next cluster was selected by adding the sampling interval to a random number. The same procedure was repeated until all three village clusters were identified, plus their sampling distribution to each cluster.

The spinning method was employed to select the direction to start the selection of households and, hence, participants. A purposive sampling procedure was employed to sample all the mothers who were found to have a child of 0–5 years in the selected village. The main goal of purposive sampling was to focus on particular characteristics of a population of interest, which enabled the principal investigator to obtain answers to research questions.

However, some limitations in representativeness must be acknowledged, as the selection of specific clusters with high agricultural activity may not fully capture the variability in socioeconomic and environmental factors across the entire SAGCOT region. The exclusion of children with chronic illnesses, genetic disorders, or severe malnutrition, while necessary for methodological consistency, might have led to the underrepresentation of certain vulnerable subpopulations. These factors are noted as potential limitations, and future studies could expand the scope to include a broader demographic and environmental range to enhance generalizability.

### 2.3 Variables

Child neuro-disability, which was the effect measured by the Malawi Child Development Tool (M-DAT), whereby a child was either medium or low development in all four measured development levels of gross, fine, language, hearing, and social, was categorized as with developmental effect. Sociodemographic factors included age (mother and child) since neurodevelopment is highly age-dependent, with critical milestones differing across developmental stages. Including age categories allows for the identification of age-specific vulnerabilities or delays, as sex and gender differences are well-documented in neurodevelopmental outcomes. Education level, thus maternal education, is a well-established determinant of child health and development; marital status is a proxy for social and economic support systems, which have been shown to influence child development outcomes. Cluster where participants are from, race of the mother, delivery terms, and maternal occupation. Maternal engagement in farming or non-farming occupations reflects exposure to occupational and environmental risks (e.g., pesticides in agricultural work) and variations in caregiving time, which can impact child development.

While the study focused on these critical variables, other potentially relevant factors, such as detailed family income, paternal involvement, or community-level factors (e.g., access to healthcare or education), were not included due to resource and logistical constraints. Though significant, these variables were beyond this study’s scope but represent promising avenues for future research. By prioritizing variables directly linked to neurodevelopment and practical feasibility, this study provides a foundational understanding of the key factors influencing child development in the SAGCOT region.

### 2.4 Determination of neuro-disability

Child neuro-disability was screened by the Malawi Child Development tool (M-DAT), which is the screening tool approved for use in Tanzania for child development assessment as it is culturally appropriate. The Malawi Child Development Tool (M-DAT) questionnaire covers four areas of child neurodevelopment: gross motor, fine motor, language and hearing, and social development. It involved children between the ages of 0 months and 6 years. The tool was developed and used in Malawi, where it was seen to be culturally perspective and acceptable; thus, following the study in Malawi, reliability was good for items remaining, with 94–100% of items scoring kappas > 0.4 for inter-observer immediate, delayed, and intra-observer testing (Autism and Developmental Disabilities Monitoring Network Surveillance Year 2008 Principal Investigators and Centers for Disease Control and Prevention, 2012).

On the grid table of obtaining the level of child neuro-disability according to the question, the child score, if the level obtained is in the white shaded zone, indicates the child may need further assessment. Scores in the light blue shaded “monitoring” zone help identify at-risk children. Professionals can give parents activities to help their child progress in these areas before the next screening. Scores in deep blue zones mean the child is doing well in these areas (Kadesjo et al., 1999).

A total score was computed for each domain by summing the number of items a child passed at the 90th percentile level for their age. The scores in each domain could range from 0 to 34. Children’s scores were classified as follows: (1) normal outcome if they performed at the ≥90th percentile level on all of the items in that domain or < 90th percentile on one or two items in the domain; or (2) impaired if they performed <90th percentile on more than two items in a domain. Infants who displayed a normal outcome in all domains were classified as normally developing, and infants who were impaired in at least one domain were classified as displaying global neurodevelopmental impairment (Kadesjo et al., 1999).

**Figure 1:**
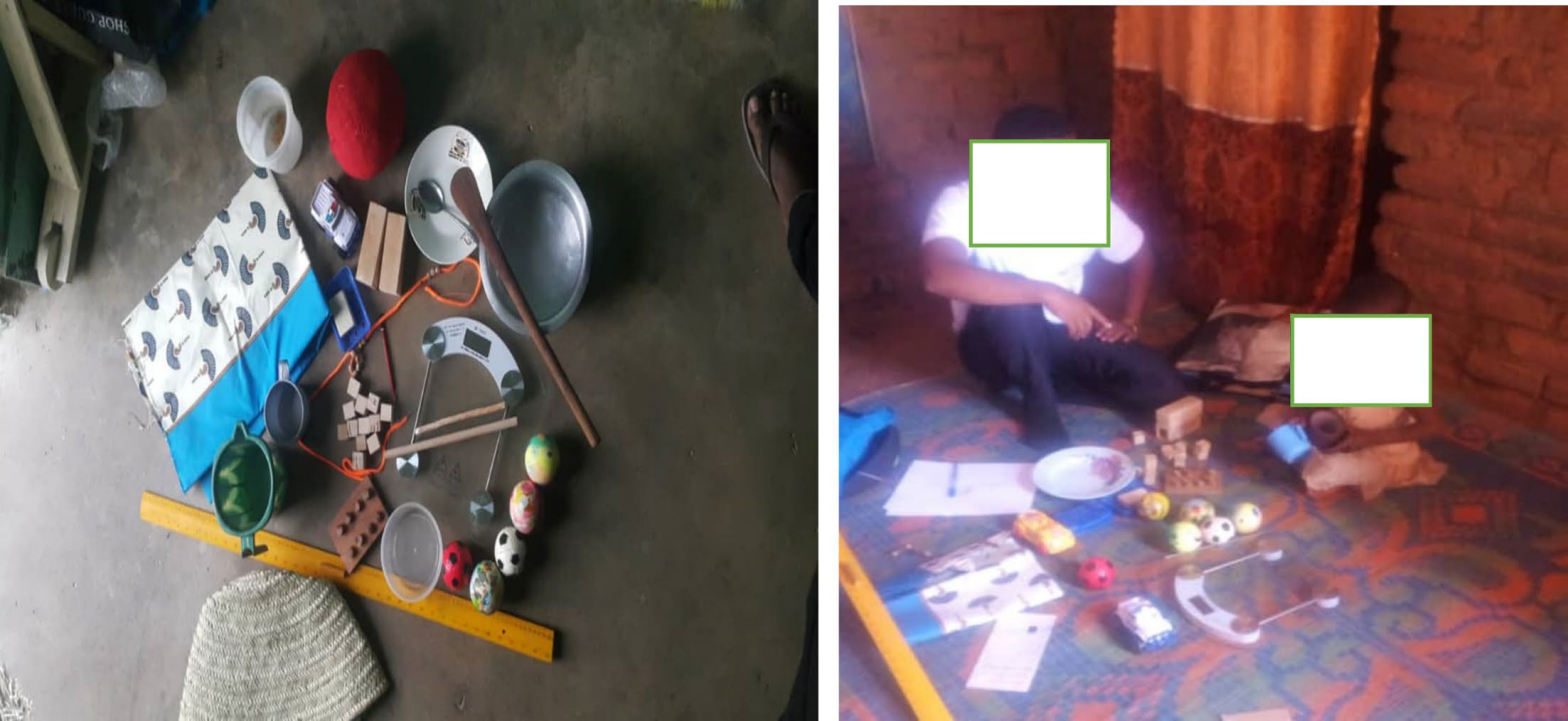
Tools for child development screening, M-DAT package.

A test of research tools and training for research assistance was conducted at Temeke Municipal Hospital, which hosted a large cohort of clinical trial studies involving children diagnosed with autism. As a result, participants clearly understood the questions, as shown in Figure 1.

### 2.5 Data collection

Data were collected by questionnaires and semi-structured questions. The questionnaire was employed to collect basic information on child-to-maternal factors, other exposure scenarios to the children, and demographic information. The author developed the questionnaire and administered it to the mothers so that information could be rendered more easily and collected.

A checklist was employed to assess other risk factors for pesticide exposure to the children in the sampled areas of the SAGCOT. It was employed to collect information on some of the practices that accelerate children’s exposure. Moreover, the checklist assisted in the assessment of the practices regarding pesticide handling, storage use, and disposal of containers, which are tantamount to exposure scenarios. The Muhimbili University of Health and Allied Sciences, Department of Pediatric Health, trained the recruited data collectors.

### 2.6 Data analysis

Data from a survey were analyzed using STATA version 18.0. Demographic characteristics, including child age, gender, marital status, occupation, age of a mother, race, and so forth, were measured to describe the sample. Bivariate logistic regression analyses were conducted to examine the relationships between independent variables and the dependent variable (in this case, global neurodevelopment). Subsequently, a multivariable logistic regression model was built with all covariates initially included to avoid omitting potential confounders. In the final model, neurodevelopmental impairment was adjusted for all studied covariates. Covariates showing a statistically significant association with neurodevelopmental outcome at a *p*-value of less than 0.20 were retained and presented in Table 1. All statistical inferences were made using 95% confidence intervals. Since neurodevelopmental impairment was a common outcome (prevalence ≥10%), modified Poisson regression with robust standard errors was used to estimate prevalence ratios (PRs) and their 95% confidence intervals (CIs). This approach is appropriate when the outcome is highly prevalent (Manji and Hogan, 2013; Kogan et al., 2009; Salari et al., 2022). The analysis focused on the association between demographic characteristics and neurodevelopmental impairment in children.

**Table 1:** Demographics factors associated with neuro-disabilities.

Variables	Gross development		Fine development		Language development		Social development		Global development disorder	
	aPR (95%, CI)	*p*-value	aPR (95%, CI)	*p*-value	aPR (95%, CI)	*p*-value	aPR (95%, CI)	*p*-value	aPR (95%, CI)	*p*-value
Age category of a child (in months)										
0–12	1		1		1		1		1	
13–48	1.08, 0.91–1.29	0.372	1.05, 0.92–1.22	0.424	1.05, 0.88–1.27	0.574	1.36, 0.87–2.15	0.194	1.08, 0.97–1.21	0.172
49–72	1.17, 0.91–1.48	0.226	0.99, 0.79–1.24	0.919	1.14, 0.88–1.48	0.305	1.22, 0.63–2.35	0.561	1.02, 0.86–1.21	0.836
Gender (Child)										
Boys	1		1		1		1		1	
Girls	1.15, 0.99–1.30	0.067	1.13, 1.02–1.26	0.025 *	1.19, 1.04–1.39	**0.014** ^*^	0.89, 0.63–1.27	0.532	1.04, 0.97–1.13	0.284
Marital status										
Not Married	1		1		1		1		1	
Married	1.23, 1.02–1.49	**0.029** ^*^	1.13, 0.98–1.32	0.102	1.20, 0.98–1.45	0.072	0.84, 0.59–1.23	0.388	1.09, 0.98–1.21	1.109
Education of a mother										
No formal education	1		1		1		1		1	
Basic education	1.07, 0.88–1.29	0.487	1.11, 0.93–1.32	0.240	1.01, 0.83–1.22	0.942	0.85, 0.57–1.29	0.450	1.11, 0.97–1.27	0.131
Collage and above	0.85, 0.57–1.25	0.406	1.09, 0.84–1.43	0.498	0.83, 0.56–1.23	0.355	0.50, 0.19–1.37	0.178	1.08, 0.90–1.29	0.417
Occupation of a mother										
Non-farming	1		1		1		1		1	
Farming	1.08, 0.94–1.25	0.274	1.03, 0.91–1.15	0.669	1.09, 0.94–1.27	0.260	0.96, 0.67–1.37	0.811	1.09, 1.01–1.19	**0.032** ^*^

aPR, Adjusted Prevalence Ratio; *p*-value, Probability value; ^*^p < 0.05.

## 3 Results

### 3.1 Sociodemographic characteristics of respondents

A total of 286 mother-to-child pair participants were enrolled in the study. Four distinct clusters were identified: Ihemi, Kilombero, Ludewa, and Mbarali, with sample population frequencies ranging from 16.8 to 32.9%. The median age of the children in the sample was 24 months, with the majority falling into the 13–48-month category. Gender distribution among children showed a slight male predominance, with 59.4% boys and 40.6% girls. The median age of mothers was 30 years, predominantly within the 21–30 age category. African ethnicity was predominant among mothers, accounting for 96.2% of the sample. Most mothers were married (72.4%) and had attained education up to the primary level (56.6%). A slight majority were engaged in non-farming occupations (56.3%). Table 2 shows the sociodemographic information.

**Table 2:** Sociodemographic information.

Variables	Frequency (*n* = 286)	Percentage (%)
Cluster		
Ihemi cluster	94	32.9
Kilombero cluster	84	29.4
Ludewa cluster	48	16.8
Mbarali cluster	60	20.9
Age of a child (Median, IQR)	24 [13.9–38.4]	
Age category of a child (in months)		
0–12	66	23.4
13–48	187	65.4
49–72	33	11.5
Gender (Child)		
Boys	170	59.4
Girls	116	40.6
Age of a mother (median, IQR)	30 [25–33]	
Age category of a mother (in years)		
18–20	72	25.2
21–30	173	60.5
31 and above	41	14.3
Race (Mother)		
African	275	96.2
Arabic	9	3.1
Asia	2	0.7
Marital status		
Married	207	72.4
Co-habiting	34	11.9
Divorced	3	2.5
Widow	4	1.4
Single	34	11.9
Level of education		
No formal education	50	17.5
Primary level	162	56.6
Secondary level	54	18.9
Collage and above	20	7.0
Occupation of a mother		
Non-farming	161	56.3
Farming	125	43.7
Delivering terms		
Full gestation	248	86.7
Not full gestations	38	13.3

### 3.2 Prevalence of neuro-disabilities

Neuro-disability was assessed across the four dimensions: gross motor, fine motor, language and hearing, and social development. With gross development, 27.3% of participants were classified as developed, while 72.7% were classified as less developed. Fine development: 19.6% were developed, while 80.4% were less developed. Language and hearing development: 30.1% were developed, while 69.8% were less developed. Social development: 67.8% were developed, while 32.2% were less developed. Another instance of global neurodevelopmental impairment is that 11.2% were developed, while 88.8% were less developed, whereas the confidence interval for less developed is 84.6–92.2%. Overall, the data suggests that in this sample, a significant portion of individuals are classified as less developed across various domains of development (Table 3).

**Table 3:** Prevalence of neuro-disability under the measured spectrum of development.

Variable	Developmental level	Frequency (*n*)	Percentage (%)	95% CI
Gross development	Developed	78	27.3	
	Less developed	208	72.7	67.2–77.8%
Fine development	Developed	56	19.6	
	Less developed	230	80.4	64.3–75.2%
Language and hearing development	Developed	86	30.1	
	Less developed	200	69.8	64.3–75.2%
Social development	Developed	194	67.8	
	Less developed	92	32.2	26.8–37.9%
Global neuro-development	Developed	32	11.2	
	Less developed	254	88.8	84.6–92.2%

### 3.3 Cross-tabulation of variables

We conducted a cross-tabulation of participant clusters and autism development spectrum to pinpoint the areas with the highest prevalence in the southern arc of Tanzania. With the global neurodevelopment, Ludewa had a significant proportion (91.7%) of children who exhibited less development in all domains compared to Mbarali, which had only (85.0%). Regarding gross development, children in Ihemi showed a higher prevalence of neurodevelopmental effects (75.5%) than other areas, while Mbarali had fewer children with developmental challenges (Refer to Figure 1).

Conversely, regarding language and hearing development, Ihemi had disproportionately more children with developmental challenges (75.5%), whereas Mbarali had fewer cases (55.0%). Similarly, in social development, Ihemi exhibited a notable prevalence of children with limited social development (40.4%), whereas the Ludewa cluster had a lower incidence of such cases (16.7%) (Refer to Figure 1).

### 3.4 Demographics factors associated with neuro-disabilities

**Figure 2:**
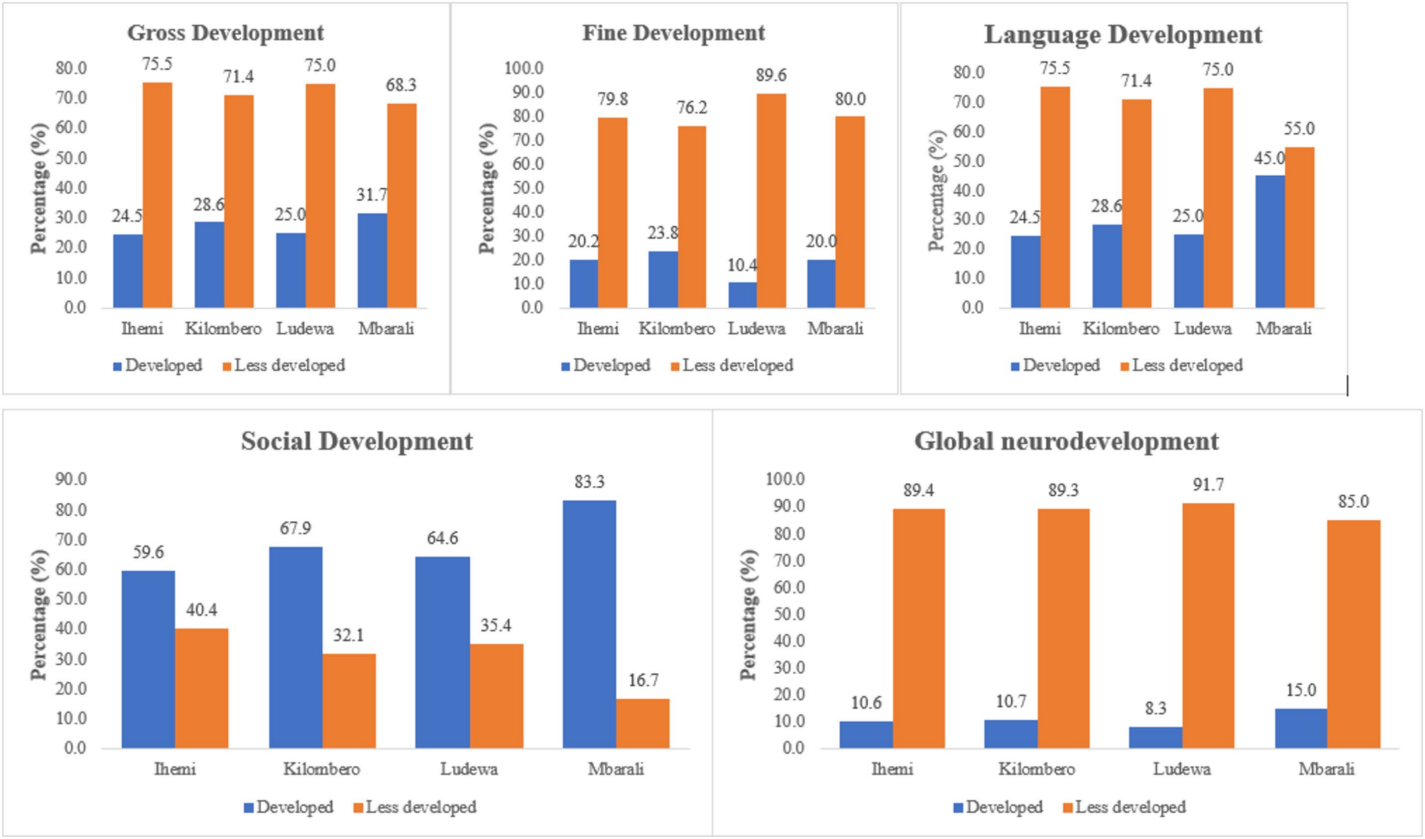
Cluster distribution of neuro-disabilities.

The table presents adjusted prevalence ratios (aPR) with 95% confidence intervals (CI) and corresponding *p*-values for various developmental domains in children, namely gross development, fine development, language development, social development, and global developmental disorder. These domains are analyzed across different demographic variables, including age category, gender, marital status, education level of the mother, and occupation (Figure 2).

No significant differences in gross, fine, language, social, or global development existed between children aged 0–12 months (reference group) and older children. However, children aged 13–48 months exhibited a small effect in gross development (aPR: 1.08, 95% CI: 0.91–1.29, *p* = 0.372) compared to the reference group. Similarly, children aged 49–72 months showed a slightly larger, though still small, effect in gross development (aPR: 1.17, 95% CI: 0.91–1.48, *p* = 0.226). Neither difference was statistically significant, suggesting limited age-related variability in these domains.

Female children demonstrated significantly higher adjusted prevalence ratios for fine motor development (aPR: 1.13, 95% CI: 1.02–1.26, *p* = 0.025) and language development (aPR: 1.19, 95% CI: 1.04–1.39, *p* = 0.014) compared to boys. These findings indicate small to moderate effect sizes and reflect a meaningful advantage for girls in these developmental domains. No significant gender differences were observed in gross development, social development, or global developmental disorder.

Married mothers had a significantly higher adjusted prevalence ratio for gross development (aPR: 1.23, 95% CI: 1.02–1.49, *p* = 0.029) than unmarried mothers, indicating a small effect size. While not significant, there was also a trend toward a higher prevalence of language development delays among children of married mothers (aPR: 1.20, 95% CI: 0.98–1.45, *p* = 0.072). No significant differences were observed in other developmental domains across marital status.

The education level of the mother did not significantly impact any developmental domain. Mothers with no formal education, basic education, or college and above had similar prevalence ratios across all domains, suggesting that factors beyond maternal education may influence child development in this study population.

Mothers engaged in farming showed a small but statistically significant effect size for the global developmental disorder (aPR: 1.09, 95% CI: 1.01–1.19, *p* = 0.032) compared to those in non-farming occupations. This underscores the potential role of occupational exposures or related stressors in contributing to neurodevelopmental risks. No significant occupational differences were observed in gross, fine, language, or social development (Table 1).

## 4 Discussion

The findings from this study align with existing literature on neuro-disability, highlighting significant developmental challenges within the sample. The high prevalence of less developed classifications across gross, fine, language and hearing, and social development domains is consistent with research indicating the pervasive nature of neurodevelopmental impairments. For instance, similar studies (Autism and Developmental Disabilities Monitoring Network Surveillance Year 2008 Principal Investigators and Centers for Disease Control and Prevention, 2012; Chaste and Leboyer, 2012; Fombonne, 2010; Huerta et al., 2012; Isaksen et al., 2012; Kadesjo et al., 1999; Salimiha et al., 2018) in other regions have reported comparable developmental delays and impairment rates, emphasizing the need for targeted interventions and support services for affected individuals. Moreover, the substantial proportion of participants classified as having global neurodevelopmental impairment echoes findings from global studies, suggesting a widespread need for comprehensive assessment and intervention strategies to address the multifaceted nature of neuro-disability.

Recent advancements in managing neurodisabilities emphasize holistic frameworks, early detection, personalized care, and societal integration (Zaneva et al., 2024). Key changes include integrated biopsychosocial models addressing the multifaceted nature of neurodisabilities, aligning with findings linking maternal occupation and marital status to developmental outcomes. Diagnostic and screening innovations enable earlier, more accurate identification of delays, supporting targeted interventions, particularly in underserved regions like SAGCOT. A growing focus on individualized interventions tailors care to unique needs, as seen in gender-specific findings. Strategies emphasizing social and environmental determinants focus on occupational safety and minimizing exposure to toxins. Inclusive policy frameworks promote equitable education and healthcare, addressing disparities like those in Ludewa and Ihemi. Interdisciplinary collaboration ensures evidence informs practice. This study underscores the need for proactive strategies, regional interventions, and policies to improve outcomes for children with neurodisabilities.

However, this study pointed to the prevalence in accordance with various dimensions, whereby gross development had many children with the disorder compared to other dimensions, and language and hearing development had fewer. This is quite similar to most of the studies conducted on autism (Autism and Developmental Disabilities Monitoring Network Surveillance Year 2008 Principal Investigators and Centers for Disease Control and Prevention, 2012; Awadu et al., 2022; Bakare et al., 2012; Baron-Cohen et al., 2009; Boyle et al., 2011; Chaste and Leboyer, 2012), whereby gross was prominent. This can be explained by the method used to screen the disorder, which has made it an easier way to assess it and thus easy to observe compared to other dimensions. In our study area, most of the children had gross development disorders, which can be attributed to inadequate everyday physical activities like walking, running, throwing, lifting, and kicking children since most of their parents are busy with income generation.

The cross-tabulation of participant clusters and neuro-disability in the southern arc of Tanzania revealed notable variations in neurodevelopmental challenges across different regions. Ludewa had the highest proportion of children exhibiting less development in all domains, indicating significant developmental disparities compared to Mbarali. Ihemi stood out with a higher prevalence of neurodevelopmental effects in gross motor, language, hearing, and social development domains, underscoring the need for targeted interventions in this area. These findings align with previous research emphasizing geographical disparities in neurodevelopmental outcomes (CDC, 2023), highlighting the importance of region-specific approaches to address developmental challenges.

Regarding age category, while no significant differences were observed in children aged 0–12 months compared to the reference group, girls aged 13–48 months demonstrated higher prevalence ratios for fine and language development than boys. We compared with other studies that pointed out that age is one factor that exacerbates neuro-disability among children. A study conducted in the UK (Xu et al., 2023) pointed out that ASD prevalence estimates varied widely across all sites in accordance with the age groups. The variations might be due to the difference in methodology and the deviations among the study participants, whereby this study had more diversity since it collects information from the community. Given the observed differences in developmental outcomes between girls and boys aged 13–48 months, further research is warranted to explore potential underlying factors contributing to these disparities. This could involve conducting longitudinal studies to examine developmental trajectories and identify specific areas of concern for each gender.

Our findings point out that girls are more likely to develop fine motor and language developmental disorders compared to men. This aligns with the majority of studies, which suggest that neuro-disabilities tend to affect female children more than boys (Bakare et al., 2012; Hoshino et al., 1982). However, the findings of some studies differ from our findings, such as a study conducted in the UK (Hoshino et al., 1982), which reported significantly higher ASD prevalence estimates among boys compared to girls across all 14 ADDM sites, with male-to-female prevalence ratios ranging from 2.7 in Utah to 7.2 in Alabama.

The gender proportions in other studies were not as balanced as in this study, which forms a basis for observed variations. Notably, in this study, most boys were found in areas with a higher prevalence of risk factors compared to girls. These variations in gender proportions among study participants may influence the prevalence estimates of neuro-disability disorders.

Boys in this study were more frequently found in areas with higher environmental risks, such as exposure to agricultural pesticides, potentially mitigating the observed gender disparities. Variations in gender proportions across studies may also contribute to differences in prevalence estimates, emphasizing the importance of ensuring gender balance in future research. Future studies should carefully consider gender balance in participant recruitment to ensure representative samples and enhance the validity and generalizability of findings. Thus, longitudinal studies are needed to explore the developmental trajectories of boys and girls, examining potential environmental, genetic, and sociocultural influences on neurodisabilities.

Our findings indicate that married mothers were at a higher risk of having children with gross developmental delays compared to unmarried mothers, which is relatively consistent with the majority of studies from the UK and some African countries, such as Nigeria (Autism and Developmental Disabilities Monitoring Network Surveillance Year 2008 Principal Investigators and Centers for Disease Control and Prevention, 2012; Chilipweli et al., 2021; Boyle et al., 2011; Huerta et al., 2012; Salimiha et al., 2018), which also show that married women are more likely to have children with neuro-disabilities. This can be attributed to traditional gender roles in Africa, where women are often exposed to factors contributing to neuro-disabilities, such as chemicals used in washing clothes and cleaning homes. Additionally, the recent SAGCOT policy, aimed at increasing agricultural production, has led many married women to engage in strenuous work to raise family incomes and reduce dependence, further exposing them to risk factors.

In the SAGCOT region, the push for increased agricultural production may disproportionately burden married women with physical labor and exposure to chemicals, contributing to neurodevelopmental risks. This highlights the potential role of family structure and support systems in fostering optimal child development and addressing sociocultural norms and gender roles that may contribute to these disparities.

Notably, recent developments (Zeidan et al., 2022) emphasize the integration of biopsychosocial models, which recognize the interplay of biological, psychological, and social factors in shaping neurodevelopmental outcomes. This paradigm shift moves beyond isolated medical or environmental explanations, advocating for holistic assessments that capture the complexity of neurodisabilities. For example, incorporating family dynamics, environmental exposures, and community-level factors into screening and intervention programs aligns with our study’s emphasis on demographic and occupational influences, such as the higher prevalence of global developmental disorders among children of farming mothers.

More efforts should focus on promoting gender equality, empowering women, and challenging traditional roles that may expose women to factors contributing to neuro-disabilities. Moreover, measures should be implemented to mitigate potential risks associated with increased exposure to chemicals and other environmental factors in the workplace and at home. Health education programs should be implemented to raise awareness among women about the potential risks of certain activities or exposures during pregnancy and early childhood.

Furthermore, the mother’s education level did not significantly impact developmental outcomes, suggesting that factors beyond maternal education may influence child development. Similarly, while mothers engaged in farming occupations showed higher prevalence ratios for a global developmental disorder, the occupation did not significantly affect other developmental domains, indicating the need to further explore occupational influences on child development.

## 5 Conclusion

The research effectively delineates neuro-disability across the four dimensions in the southern corridor of Tanzania. High prevalence rates of less developed classifications across various domains emphasize the pervasive nature of neurodevelopmental impairments. Notably, notable disparities exist across different geographical areas, underscoring the need for targeted interventions. Additionally, gender and marital status were identified as significant factors influencing developmental outcomes, warranting further research and tailored interventions. Promoting gender equality, empowering women, and mitigating environmental risks are essential to fostering optimal child development. This study contributes to the understanding of neuro-disability and emphasizes the importance of comprehensive assessment and intervention strategies to address developmental challenges effectively through their multifaceted factors to enhance child development and overall wellbeing.

## Data Availability

The raw data supporting the conclusions of this article will be made available by the authors, without undue reservation.
